# Straw pyrolysis for use in electricity storage installations

**DOI:** 10.1016/j.heliyon.2024.e30058

**Published:** 2024-04-25

**Authors:** Jerzy Chojnacki, Jan Kielar, Jan Najser, Jaroslav Frantík, Tomáš Najser, Marcel Mikeska, Błażej Gaze, Bernard Knutel

**Affiliations:** a VSB-Technical University of Ostrava, CEET, ENET Centre, 17. Listopadu 15, 708 00, Ostrava-Poruba, Czech Republic; b Institute of Technology and Life Sciences—National Research Institute, Falenty, Al. Hrabska 3, 05-090, Raszyn, Poland; c Institute of Agricultural Engineering, Wrocław University of Environmental and Life Sciences, 51-630, Wroclaw, Chełmońskiego 37a, Poland

**Keywords:** Pyrolysis products, Electricity storage installation, Biochar, Energy balance, Straw, Carbon sequestration

## Abstract

A concept has been proposed for an installation designed to store excess electricity periodically occurring on the grid. Excess electricity will be used for straw pyrolysis. The main pyrolysis product, gas, will be used to generate electricity using a combustion generator to feed back power into the grid during periods of shortage. The resulting biochar from the pyrolysis can be introduced into the soil to improve soil quality and play a significant role in carbon sequestration. The system uses an electrically heated reactor with a screw conveyor.

To preliminarily assess the feasibility of this system, experiments were carried out using wheat straw at temperatures of 300, 400, 500, 600, and 700 °C for the pyrolysis reactor. The resulting gas-to-feedstock mass ratio ranged from 29.04 % at 300 °C to 52.7 % at 700 °C reactor temperature, the biochar mass yield ratio to feedstock varied from 39.41 % to 27.36 % (at 700 °C), and the pyrolysis liquid ranged from 31.55 % to 27.36 % (at 700 °C). The pyrolytic liquid contained a high water content relative to its mass, reaching up to 95.2 % at 700 °C, rendering it less suitable as an energy feedstock.

At a reactor temperature of 700 °C, the energy value of the gas produced from the feedstock was twice that of the electricity used for the pyrolysis process. These results suggest the feasibility and operation of the proposed installation.

## Highlights

• Straw pyrolysis as a form of Power-to-Gas-to-Power technology.
• Renewable source of electricity and heat.
• Biochar for carbon soil sequestration.
• Synthesis gas purification and refining.
• Will water in a pyrolytic liquid allow its energy use?

## 1 Introduction

A substantial amount of waste biomass remaining from farm production is often developed through composting and utilized as natural fertilizer for the soil. Left to biodegrade naturally, biological waste decomposes under the influence of aerobic and anaerobic bacteria, resulting in the emission of greenhouse gases (mainly carbon dioxide and methane) that contribute to atmospheric and environmental pollution [1,2]. To preserve the valuable energy properties of agricultural waste biomass, it can be converted into methane through biogas plants for wet biomass [3], while dry biomass, commonly straw, can be burned in heating boilers [4,5]. Although burning dry biomass in heating boilers is the most straightforward method to extract energy from it, this process has negative environmental implications, including the release of dust clouds and toxic gases [6,7].

The worldwide trend towards clean energy production has driven the advancement of photovoltaic systems and wind power plants. Numerous photovoltaic and wind power facilities are situated near farms and many farmers own such installations. Given that electricity generation from these sources depends on factors like the season, time of the day, and atmospheric conditions, imbalances between electricity production and consumption (due to immediate current demand) can emerge [8]. A potential solution to mitigate the issue of surplus or deficit electricity during different time periods is to store the excess energy and deploy it when electricity demand exceeds supply [[9], [10], [11]] One of the possible solutions is the "Power-to-Gas-to-Power (P2G2P) technology, which involves converting electricity into hydrogen and oxygen by electrolysis, of the raw material, i.e., water, enabling subsequent electricity generation using hydrogen cells [9,10,12]. A feasible approach, approach, especially in agriculture, is the production of synthetic methane via thermochemical processes using another available feedstock - waste biomass [10].

In the context of agricultural farms, a more environmentally aligned approach to stabilize grid electricity voltage involves using surplus electricity to pyrolyze waste biomass. This process has the advantage of yielding multiple products that can be stored and employed for electricity generation, heating, and agricultural applications [13,14]. Biochar, obtained by pyrolysis of biomass, is a valuable product due to its numerous organic, energetic and chemical properties. The temperature of pyrolysis and the composition of the feedstock affect the mineral content of biochar [[15], [16], [17], [18], [19]]. Increased mineral content could result from higher pyrolysis temperatures [15,18,19]. The introduction biochar in agriculture can increase soil microbial activity, reduce soil density, and in affect nutrient and water retention. resulting in improved them to plant availability ][[20], [21], [22], [23]]. The introduction of biochar into the soil can not only increase yields but also sequester atmospheric carbon. Its production can be an important motivating target for biomass pyrolysis on farms, especially since farmers have the raw material locally. In this scenario, biomass pyrolysis seems to be an effective solution for electricity recovery, heat generation, and biochar production.

Biomass pyrolysis gas, once purified, can be stored and subsequently burned in farm heating boilers, but it can also serve as fuel for internal combustion engines or turbines that power generators [24,25]. This approach enables the generation of both electricity and heat from the gas. Bio-oil obtained from pyrolysis can be utilized similarly to pyrolysis gas.

The most common waste biomass from agricultural production that is well-suited as an energy source and for biochar production is straw left over from grain harvesting. Large-scale industrial plants are already operated for the pyrolysis of waste straw [[26], [27], [28]]. In these installations, the necessary heat for biomass pyrolysis is generated either through partial combustion of the raw material or by direct combustion of the gaseous and liquid products derived from the process. The necessary heat can also originate from burning external energy sources, such as oil or gas from fossil fuels [29]. Tailored installations are designed to meet the requirements of farmers, encompassing pyrolysis of waste from poultry production, grass straw, and wood waste [30,31]. Plant capacities range from 80 kg d^−1^ and can reach up to one ton daily.

By utilizing electricity-powered reactors during periods of excess energy in the grid, performing pyrolysis of agricultural biomass waste on farms could lead to improved electricity management, as surplus energy could be stored for use during shortages. The stored pyrolysis gas, and possibly bio-oil as well, could function as an energy reservoir [32]. This approach would also mitigate environmental pollution stemming from flue gas produced by farm furnace boilers. It might prove to be more environmentally friendly than the utilization of heat pumps for heating farm buildings. Preliminary simulation calculations lend support to this conclusion [33].

Screw reactors equipped with electrically heated chambers for biomass pyrolysis allow simple control over the amount of supplied biomass and the process temperature. They facilitate stable process conditions [34,35] and are well-suited for energy conversion in pyrolysis [29,35].

The research aimed to formulate a concept for installing an electric reactor to pyrolyze waste agricultural biomass, such as straw, to store electricity using the gas produced during pyrolysis. Therefore, the research compares the energy consumption in pyrolysis with the energy content of the pyrolysis products, as well as to create a mass balance and energy analysis of these products and to evaluate changes in their chemical composition.

The research assumed that an advantage of implementing pyrolysis in an electricity storage system would be producing additional thermal energy and biochar. Specifically, the research also aimed to determine the amount of biochar, its technical parameters, and its chemical composition.

## 2 Materials and methods

### 2.1 Installation concept

**Fig. 1:**
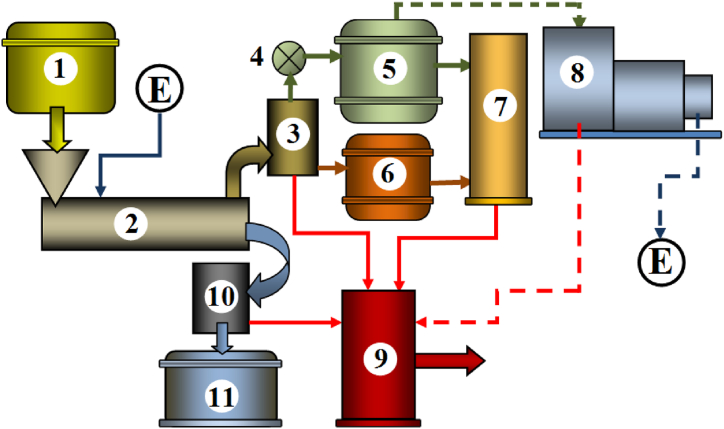
Electricity storage installation concept: (1) raw material tank, (2) screw reactor, (3) gas products cooler, (4) pump for pumping gas to the tank, (5) gas tank, (6) bio-oil tank, (7) heating boiler, (8) combustion power generator, (9) heat storage - hot water tank, (10) biochar cooler, (11) biochar storage.

The concept of an installation designed to store electricity using straw pyrolysis, carried out using an electric screw reactor, is shown in Fig. 1.

The products exiting the reactor consist of hot biochar and a heated mixture of substances in a gaseous state containing pyrolysis gas and bio-oil. To facilitate storage and, at other times since pyrolysis time, the use. of these gasified products as energy sources, the design involves cooling them in a cooler (3) and then pumping the gas separately via a pump (4) into a gas tank (5). The bio-oil released during the cooling process could be stored in a subsequent tank (6). The installation layout also incorporates a reservoir for storing thermal energy destined for consumers (9). The heat supplied to this reservoir originates from the cooling process of pyrolysis products, the heating boiler (7) where bio-oil and gas are combusted, and the gas combustion within the electricity generator (8).

### 2.2 Test stand

**Fig. 2:**
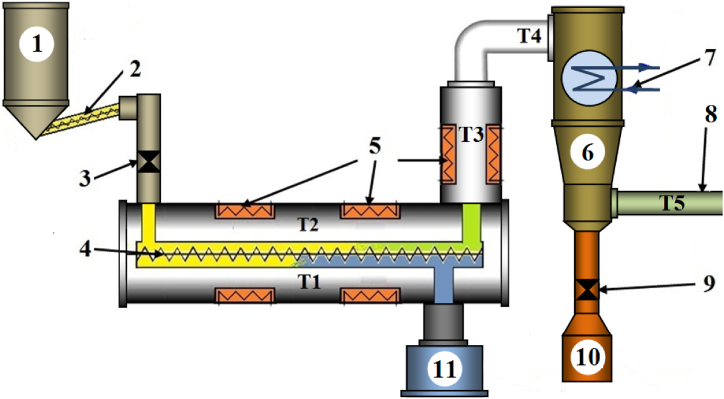
Scheme of the test reactor with equipment: (1) raw material container, (2) raw material screw dispenser, (3) valve, (4) reactor screw conveyor, (5) electric heaters of the reactor, (6) gaseous substance cooler, (7) pipes (outlet and inlet) of cooling water, (8) gas outlet, (9) oil drain valve, (10) container for oil, (11) container for biochar.

A test stand with a screw reactor was prepared for the continuous pyrolysis of biomass. A schematic illustrating the reactor's operation and its additional equipment is shown in Fig. 2.

The test stand was part of the installation and consisted of an electrically heated pyrolysis chamber with a screw conveyor (4), a cooler for the gaseous product mixture (6), a bio-oil tank (10), and a biochar tank (11). The raw material (biomass) was transferred to the reactor from tank (1) via a screw dispenser (2) with a fixed capacity. A valve (3) prevented the backflow of pyrolysis gases after the feedstock batch was completed in the tank. The reactor was heated by three electric heaters (5) with a total input of 8.9 kW. Thermocouples measuring the reactor's temperature (T1, T2, T3) were placed on the outer surface of the reactor body near the heating elements. The pyrolysis device's control system enabled the regulation of the process temperature, maintaining it at the same level across all three measuring points (T1, T2, T3). The gas cooler (6) was constructed with piping supplied with tap water, forming a "water jacket" that surrounded the pipe chamber containing gaseous substances, cooling them and allowing the separation of bio-oil from the gas. Using thermocouples, the temperature of the gas entering the cooler from the reactor (T4) and the temperature of the gas after bio-oil separation as it flowed out of the cooler (T5) were measured. The liquid condensate from the gas was gathered in the lower section of the cooler (6) and subsequently intermittently directed through valve (9) into tank (10).

### 2.3 Materials

Due to the widespread cultivation of wheat as a grain worldwide, wheat straw in granular form was selected as the test material for the study. Previous investigations into the pyrolysis of wheat straw were conducted using fixed-bed thermal reactors [14,36] and flow reactors [18,37,38]; however, these studies did not analyze wheat straw pyrolysis as a method of electricity accumulation.

**Fig. 3:**
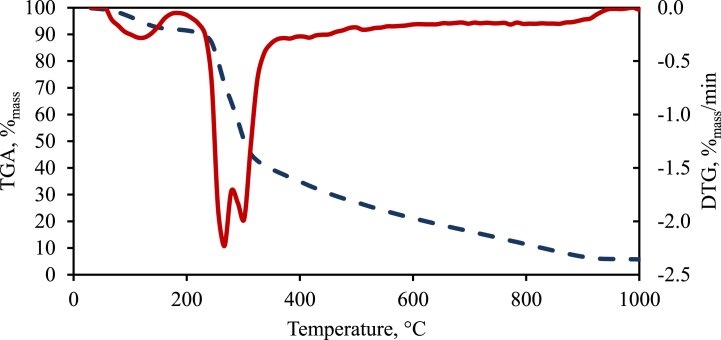
Thermogravimetric analysis of raw wheat straw pellet.

For an initial assessment of the impact of temperature on the mass reduction of the prepared raw material and the rate of mass loss, its thermogravimetric decomposition was conducted (under a nitrogen atmosphere) using a TGA 701 analyzer. Based on the results from this analysis, the graph shown in Fig. 3 was drawn up.

This graph served as a preliminary source of information regarding the changes taking place in wheat straw subjected to thermal decomposition. From the TGA and DTG curves, the pyrolysis temperature values optimal for the highest gas release rates were assessed.

Since the pellets contained a small amount of moisture, the entire prepared batch of granulate underwent drying in a Binder FP 400 dryer to remove the water content conducting the experiments. The fundamental physical parameters of the wheat straw pellet are shown in Table 1.

**Table 1:** Parameters of dry wheat straw pellet.

Diameter, mm	Moisture before drying, %_mass_	Moisture after drying, %_mass_	Density, kg·m^−3^	Bulk density, kg·m^−3^
10.42 ± 0.16	7.08 ± 0.09	0.52 ± 0.18	1126.55 ± 32.67	575.07 ± 7.33

The raw material used in the study was subjected to ultimate and proximate analysis. Ultimate analysis was made using a LECO CHSN628 analyzer. Proximate analysis of the raw material was measured by TGA (thermogravimetric analysis) using a TGA 701 analyzer. The results of the measurements are shown in Table 2.

**Table 2:** Parameters of wheat straw.

Ultimate analysis, %_mass_					Proximate analysis, %_mass_				Higher heat. val. (HHV), MJ·kg^−1^
C	H	N	S	O	Ash	Fuel total	Volatile fuel	Solid fuel	
47.40 ±0.87	5.72 ±0.91	0.33 ±0.06	0.12 ±0.01	42.98 ±1.09	6.8 ±0.5	93.2 ±0.5	70.6 ±1.8	22.6 ±2.2	18.307 ±0.705

In order to evaluate the effect of reactor temperature and the amount of electricity consumed on the results obtained, the pyrolysis tests were conducted at reactor temperatures of 300, 400, 500, 600, and 700 °C. The weighted mass of the batch of pellets remained consistent in all experiments, i.e., 6.0 kg. The biomass within the reactor was transported at a constant rate using a screw conveyor set at 0.45 rpm. The mass flow rate of pellets in the reactor was 0.098 ± 0.002 kg min^−1^. At these settings, within 8 h of operation per day, the reactor was able to process 47 kg of biomass (i.e. about 1 ton of biomass within a month).

### 2.4 Pyrolysis products

The mass of biochar was assessed after the completion of each experiment and cooling the reactor based on the weighed mass of the product collected in the biochar container (11) Fig. 2.

Upon exiting the cooler, the synthesis gas passed through the impingers, where any residual oil was collected. The capacity of the pyrolysis liquid was evaluated at the end of each pyrolysis experiment by weighting the oil container (10), and the impingers were the gas was cleaned and subtracting the mass of these containers weighted before experiment.

Gas analysis was performed online from the gas line downstream of the impingers, using a probe. A portable synthesis gas analyzer, Gas 3100 (Hubei Cubic-Ruiyi Instrument Co. Ltd, 2017), was employed to analyze the synthesis gas obtained from the probe. This analyzer identified and recorded the volume percentages of gases including CO, CO_2_, CH_4_, H_2_, and O_2_. The total mass of gas separated from the test material during the experiment, at a specific temperature, was calculated by determining using equation (1):

(1) $$M_{g}=M_{s}\;-\;M_{c}\;-\;M_{o}$$

where: M_g_ - the mass of gas obtained by pyrolysis (kg); M_s_ - the mass of feedstock (kg); M_c_ - the mass of solid residue after pyrolysis biochar (kg); M_o_ - the mass of liquid (bio-oil) (kg).

### 2.5 Energy input measurements

Because the mass of the feedstock used in all experiments was always constant, the energy input consumed to perform each experiment was only due to the reactor's temperature. The electricity energy input was measured from the moment the dispenser (2) was turned on, and the valve (3) (Fig. 2) opened until the process was completed. A DK-ELVIS ETS47B1–P electrical meter, accuracy class B, was used for the measurement.

### 2.6 Statistical analysis

All test results were subjected to analysis of variance to determine the significance of the factors studied on the obtained results. Additionally, regression analysis was employed to determine trend lines and mathematical models describing these outcomes. The resulting mathematical equations were then utilized for energy and material analyses of the pyrolysis process. Statistical processing of the data was conducted using Statistica ver. 13.3 software by StatSoft and Microsoft Excel.

## 3 Results and discussion

### 3.1 Pyrolysis products

Thermal decomposition experiments of wheat straw pellets in the reactor were conducted in three repetitions for each temperature. Before the weighed batch of biomass feedstock was transferred into the reactor vessel, the vessel was filled with nitrogen. Once the reactor reached the desired temperature, the valve supplying biomass to the reactor was opened.

**Fig. 4:**
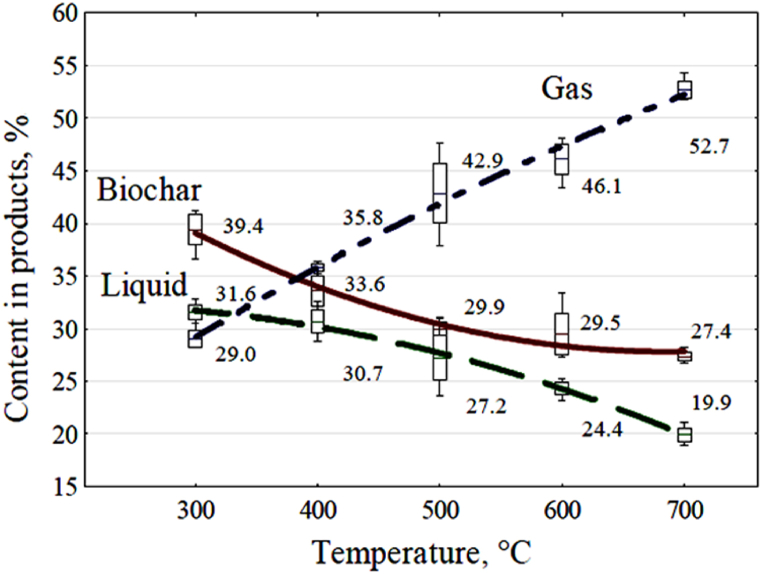
Effect of reactor temperature on the percentages of products in relation to the mass of raw material used (box plot, middle line - mean value, box delineates ± standard error, whiskers show max. value and min. value from the results).

The mass yields for each pyrolysis product were used to calculate their percentages relative to the total mass of the raw material. The results of these tests and the proportions of all pyrolysis products relative to the raw material are graphically presented in Fig. 4.

The trend lines and mathematical models, assumed to be quadratic polynomials, were selected using a statistical program. These equations depict variations in the mass yield proportions of individual pyrolysis products relative to the total mass of the raw material, as functions of the process temperature. They are represented by equations (2), (3), (4).

(2) $$M_{c}/M_{s}=60.9671-\;0.095x+0.00006919x^{2}$$

(3) $$M_{o}/M_{s}=30.2507+0.0246x\;-\;0.000057833x^{2}$$

(4) $$M_{g}/M_{s}=11.3943+0.059x$$

Where: M_c_/M_s_ - share of biochar mass yield in relation to the mass of raw material, %, M_o_/M_s_ - share of liquid mass yield to the mass of raw material, %, M_g_/M_s_ - share of gas mass yield to raw material, %, x - temperature value, °C.

The coefficient of determination R^2^ was calculate for the equations relative to the observed values. The R^2^ values for these mathematical models were as follows: for M_c_/M_s_, R^2^ = 0.9448; for M_o_/M_s_, R^2^ = 0.9419; and for M_g_/M_s_, R^2^ = 0.9926. The analysis of variance demonstrated the significance of the process temperature's impact on changes in product content.

#### 3.1.1 Gas analysis

The temperatures of the gas leaving the reactor, entering into the cooler, and the cooled gas after the bio-oil was separated are shown in Table 3. The temperature of the gas leaving the reactor was significantly lower than the temperature measured on the outer surface of the reactor, close to the electric heaters. By comparing the gas temperature with the DTG curve plotted in Fig. 3, it can be considered with some approximation that the average gas temperature at reactor temperatures of 600 and 700 °C was close to the temperature of the highest pyrolysis and gas generation rate.

**Table 3:** Temperatures of gas leaving the reactor before cooling and gas after cooling.

Temperature of reactor (T 1,T2,T3),°C	300	400	500	600	700
Gas temperature before cooling, °C	141.7 ± 11.0	185.8 ± 14.0	210.3 ± 8.5	247.1 ± 7.2	282.2 ± 6.5
Temperature of cooled gas, °C	25.4 ± 1.2	22.4 ± 0.0	19.6 ± 0.4	22.1 ± 0.3	22.8 ± 0.2

**Fig. 5:**
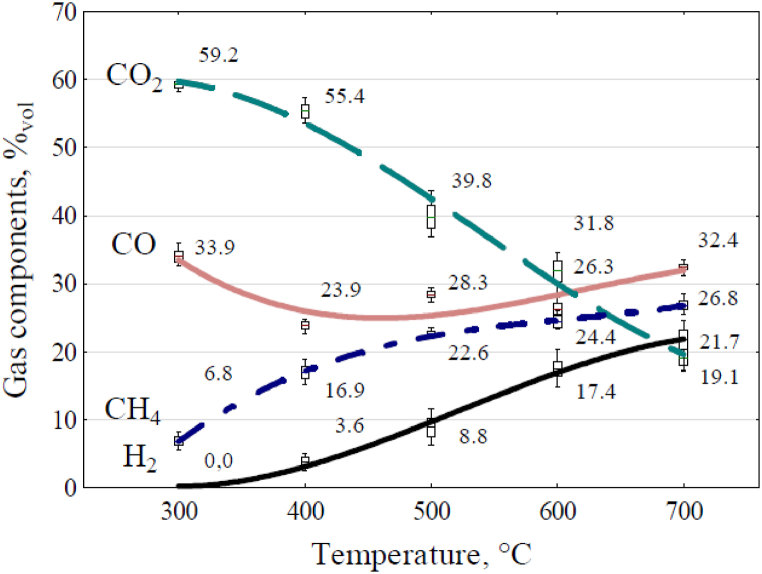
Effect of the reactor temperature of the wheat straw pellet on the percentages of CO, CO_2_, CH_4_, and H_2_ in the volumetric composition of the gas.

Using the Portable Syngas Analyser Gas 3100, gas volumetric composition data was recorded, and an analysis of the ratio of gases such as CO, CO_2_, CH_4_, and H_2_ was carried out for each complete biomass gasification cycle and various temperatures as a part of this study. The percentage values of the gases are presented in the form of a box plot (Fig. 5). The upper and lower edges of the box are ± standard deviation values, and the whiskers show the minimum and maximum values of the measurement results.

The graph in Fig. 5 shows that higher percentages of high-energy gases (i.e. methane and hydrogen) were obtained while reactor temperatures increased. Simultaneously, the carbon dioxide decreased. On the basis of the results obtained, regression equations (5), (6), (7), (8), describing the variations in the proportion of each gas in the pyrolysis gas as a function of reactor temperature were derived.

(5) $$\text{CO}=71.64571-\;0.18489x+0.00018x^{2}$$

(6) $${\text{CO}}_{2}=76.631\;-\;0.0309x\;-\;0.000070876x^{2}$$

(7) $${\text{CH}}_{4}=-\;35.957+0.1855x\;-\;0.00014x^{2}$$

(8) $$H_{2}=-\;10.5349+0.0235x+0.0000336x^{2}$$

where: CO - carbon monoxide content, %_vol_, CO_2_ - carbon dioxide content, %_vol_, CH_4_ - methane content, %_vol_, H_2_ - hydrogen content, %_vol_, x - temperature value, °C.

**Fig. 6:**
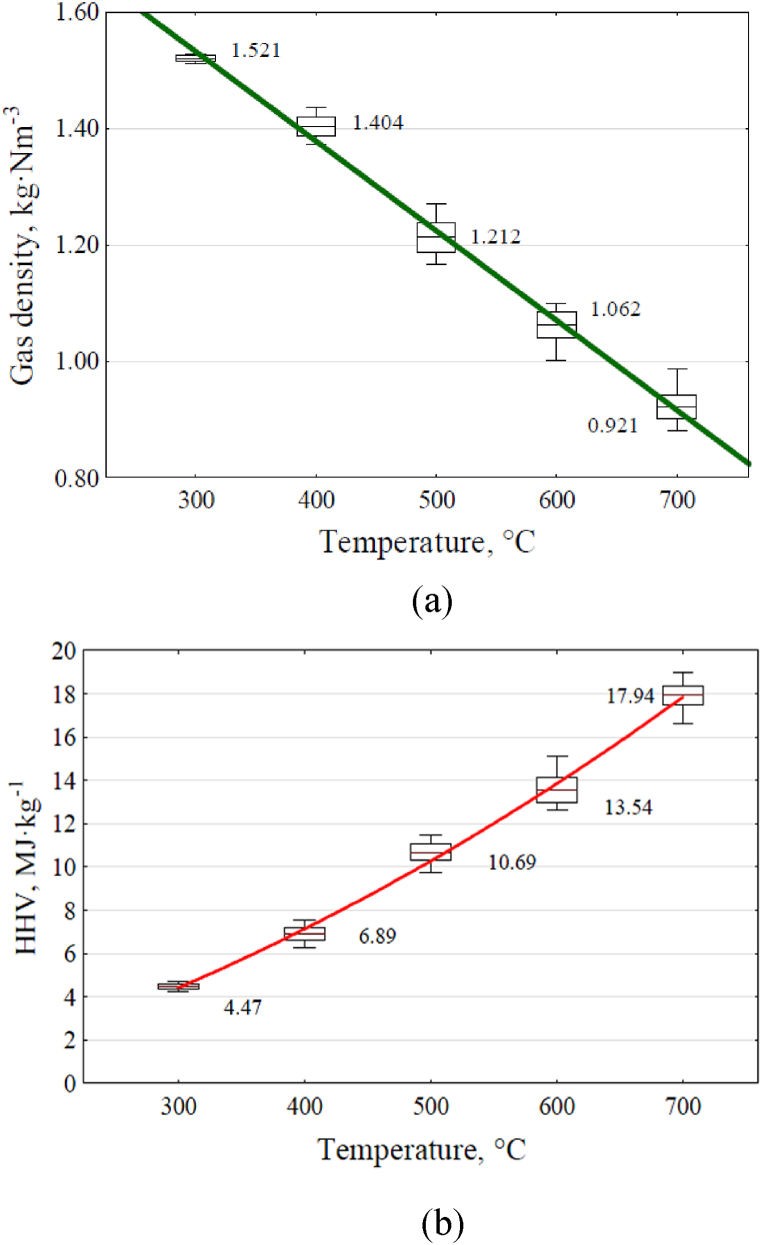
Effect of everage reactor temperature on gas properties:(a) density, (b) higher heating value.

The coefficient of the consistency of regression equations with the average values was: for CO; R^2^ = 0.5288, for CO_2_; R^2^ = 0.9824, for CH_4_; R^2^ = 0.9890, for H_2_; R^2^ = 0.9867. The volume ratios of the individual gases comprising the pyrolysis gas were utilized to calculate its density and higher heating value (HHV), which were both dependent on the process temperature. Table 4 displays the density coefficients applied in these calculations for the individual gases present in the pyrolysis gas. The influence of temperature within the reactor on alterations in gas density is depicted in Fig. 6a, while changes in the higher heating value are illustrated in Fig. 6b.

**Table 4:** Pyrolysis gas component values (higher heating value and density).

Parameter	CH_4_	H_2_	CO	CO_2_
Density, kg·m_N_^−3^	0.664^P^	0.0832^P^	1.159^P^	1.827^P^
HHV, MJ·kg^−1^	55.5^P,B^	142.1^P,^	10.9^B^	0.0

Trend lines and equations (9), (10) were determined to illustrate the influence of reactor temperature on changes in HHV and gas density.

(9) D=1.9943−0.0015x

(10) $$H=-\;0.8301+0.0132x+0.000021507x^{2}$$

where: D - gas density value, kg·m_N_^−3^, H - higher heating value, MJ·kg^−1^.

The coefficients of determination for the equations and the calculated results are: for D; R^2^ = 0.9957, for H; R^2^ = 0.9970.

#### 3.1.2 Biochar analysis

Biochar samples from individual pellet pyrolysis experiments underwent ultimate and proximate analysis. Table 5 displays the mean values along with their standard deviations obtained from these measurements.

**Table 5:** Analysis of dry biochar.

Temp. °C	C %_mass_	H_2_ %_mass_	N %_mass_	S %_mass_	O %_mass_	Ash %_mass_	Fuel total %_mass_	Volatile fuel %_mass_	Fixed Carbone %_mass_	HHV MJ·kg^−1^
300	65.67 ±1.23	4.40 ±0.35	0.84 ±0.03	0.21 ±0.01	13.69 ±2.44	16.6 ±0.4	83.4 ±0.8	26.8 ±1.1	56.6 ±0.5	26.159 ±0.403
400	68.97 ±2.38	2.83 ±1.06	0.82 ±0.01	0.20 ±0.03	6.34 ±0.76	20.2 ±1.1	79.8 ±1.1	13.6 ±0.9	66.2 ±1.1	26.745 ±0.534
500	72.50 ±1.41	2.18 ±0.55	0.83 ±0.02	0.20 ±0.03	3.19 ±1.15	21.2 ±0.9	78.8 ±0.9	10.5 ±0.6	68.3 ±1.4	27.187 ±0.378
600	73.10 ±2.91	1.20 ±0.56	0.82 ±0.07	0.21 ±0.02	2.23 ±1.01	22.1 ±1.3	77.9 ±1.3	6.9 ±0.5	71.0 ±1.5	25.935 ±0.473
700	71.73 ±1.91	1.10 ±0.67	0.83 ±0.04	0.20 ±0.03	3.17 ±1.07	22.5 ±0.6	77.5 ±0.6	7.1 ±0.2	70.4 ±0.8	24.816 ±0.126

The results of the analysis of the biochar were converted into their ratio to the initial mass of the biomass, the mass of the pellets before gasification. A mathematical formula (12) was used for this purpose:

(12) $$Y_{at}=\frac{a_{t}M_{bt}}{M_{s}}$$

where: Y_at_ is the ratio of the mass of the ingredient contained in the biochar to the mass of the raw material used, %; a_t_ is the content of the element in the biochar, %; M_bt_ is the mass of the biochar, kg; M_s_ is the mass of feedstock, kg; symbol (t) indicates the temperature of the reactor for which the calculations were performed.

**Fig. 7:**
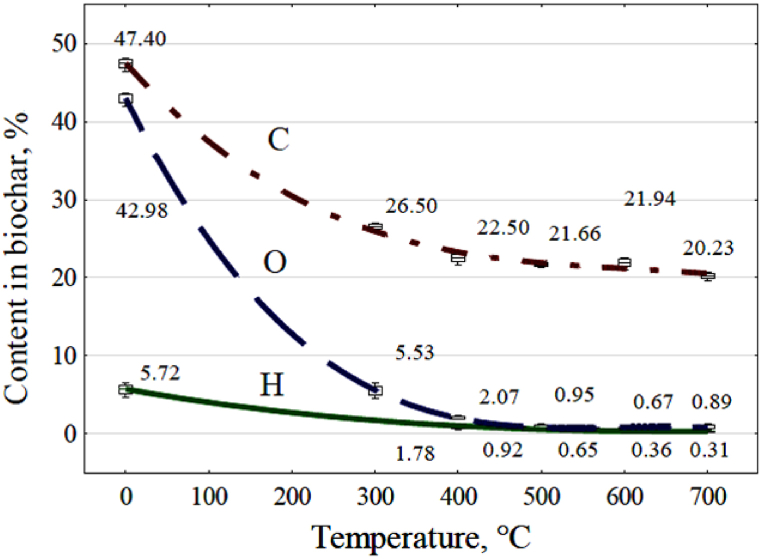
Effect of reactor temperature on changes in the percentage of the carbon, oxygen, and hydrogen concerning the mass of the raw material used.

The ultimate analysis results are presented as a graph in Fig. 7. The graph illustrates the course of change of individual components of wheat straw pellets as a function of reactor temperature during pyrolysis.

The regression equations (13), (14), (15), were also determined, describing the effect of temperature on the relationship of gas content to the mass of the pyrolyzed raw material.

(13) $$C=47.428\;-\;0.1294x+0.000224x^{2}\;-\;1.35\cdot 10^{-7}x^{3}$$

(14) $$H=5.718\;-\;0.0224x+3.26\cdot 10^{-5}x^{2}\;-\;1.68\cdot 10^{-8}x^{3}$$

(15) $$O=42.973\;-\;0.215x+0.00036x^{2}\;-\;2.0\cdot 10^{-7}x^{3}$$

where: C, H, O - are the values of the percentages of carbon, hydrogen, and oxygen in relation to the mass of pyrolyzed raw material.

**Fig. 8:**
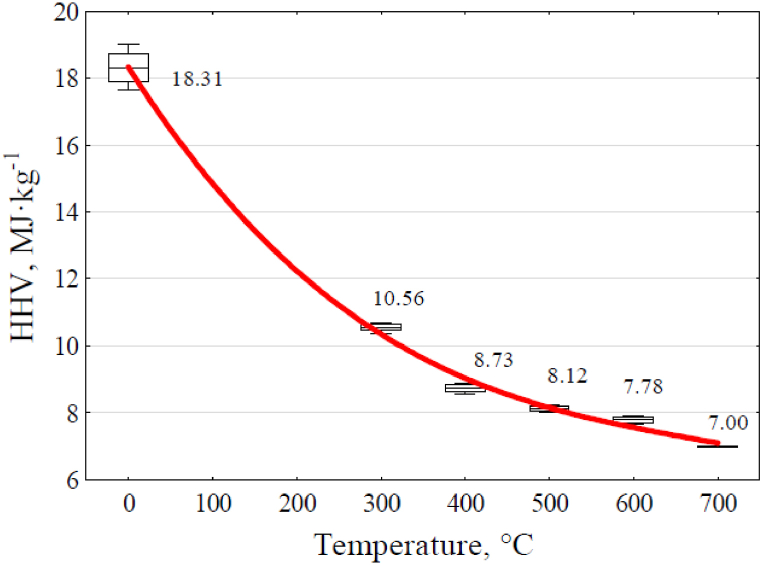
Effect of reactor temperature on value in the higher heating value of biochar, concerning the mass of the raw material used.

Fig. 8 displays the higher heating value calculated according to formula (12), relative to the mass of the pyrolyzed raw material.

Mathematical formula as a equation (16) describes the trend lines determined on the chart.

(16) $$\text{HHV}=18.322\;-\;0.0396x+5.05\cdot 10^{-5}x^{2}\;-\;2.414\cdot 10^{-8}x^{3}$$

where: HHV - the higher heating value of solid residue to raw mass, MJ·kg^−1^.

The determination coefficients were: R^2^ = 0.9977.

#### 3.1.3 Pyrolysis liquid analysis

The pyrolytic liquid obtained from the experiments contained a significant amount of water. The isolated tar-oil fraction was subjected to HHV analysis. The effect of the reactor temperature on the total balance of water and tar-oil and HHV of tar-oil fraction contained in the pyrolytic liquid is shown in Table 6.

**Table 6:** The effect of reactor temperature on the balance of water and tar-oil and HHV of tar-oil fraction.

Temperature °C	Water fraction %_mass_	Tar-oil content %_mass_	HHV_o_ of tar-oil fraction MJ·kg^−1^
300	93.3 ± 1.3	6.70 ± 1.2	28.596 ± 0.702
400	90.2 ± 0.3	9.83 ± 0.3	30.621 ± 0.986
500	78.3 ± 0.5	21.67 ± 0.5	31.344 ± 1.431
600	80.2 ± 1.0	19.80 ± 1.0	13.083 ± 0.434
700	95.2 ± 0.8	4.80 ± 0.8	18.745 ± 0.313

The percentage of water content in the pyrolytic liquid ranged from 78.3 to 95.2 % of its mass, with the lowest value achieved at 500 °C.

**Fig. 9:**
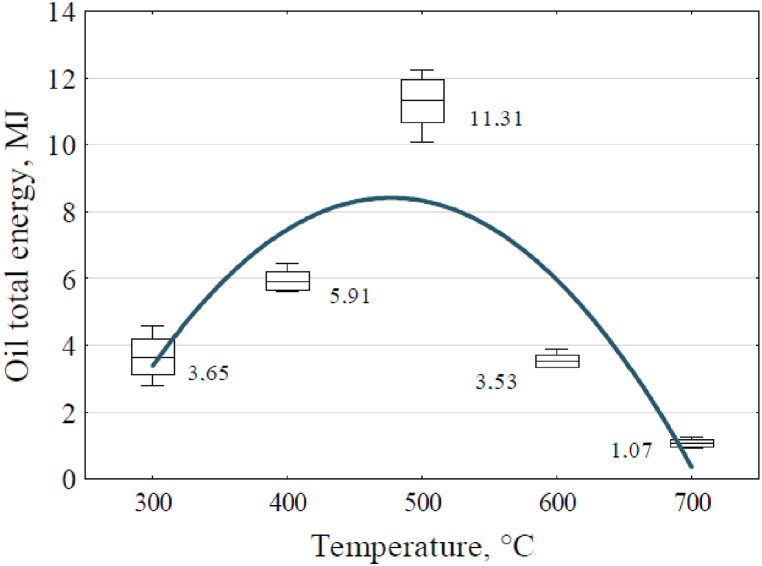
Effect of reactor temperature on the energy contained in the pyrolysis liquid.

Multiplying by each other, obtained from experiments at each tested reactor temperature, the contents of the volume of pyrolyzed liquid relative to the weight of the charge, the content of the tar oil fraction in the pyrolyzed liquid, and the HHV value of the tar oil fraction, the internal energies contained in the volumes of liquid obtained at each pyrolyzer temperature were calculated. The results of the calculations are shown, using a box plot, in Fig. 9.

The trend line of oil total energy, shown in the graph in Fig. 9, was described by equation (17):

(17) $$\text{Eo}=-23.3817+0.12976x-1.334\cdot 10^{-4}x^{2}$$

where: Eo - energy contained in pyrolysis liquid, MJ.

equation (17) coefficient of determination, R^2^, equals 0.5542. The total energy contained in the pyrolysis liquid decreased significantly in experiments above and below 500 °C.

### 3.2 Energy rating

#### 3.2.1 Electricity energy

**Fig. 10:**
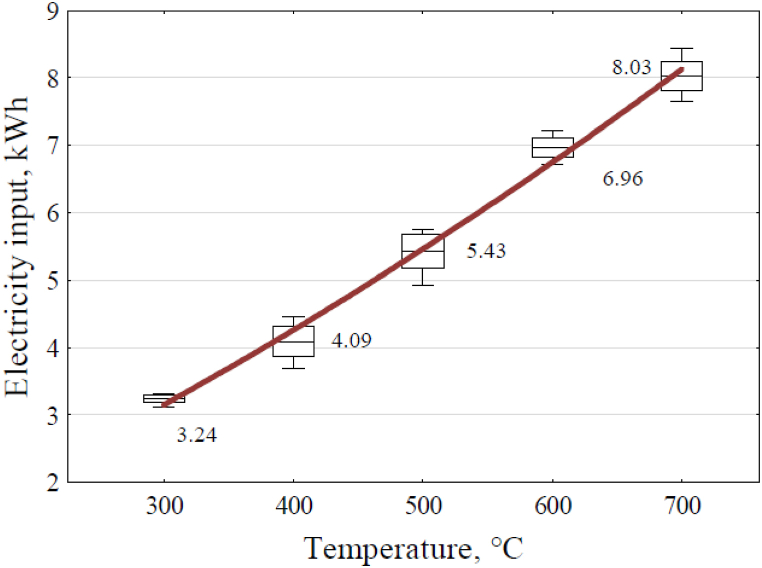
Effect of the reactor temperature on the input electricity.

The total electricity input for the pyrolysis process of 6 kg of wheat straw pellet, determined from the measurements, is shown in the graph in Fig. 10.

Equation (18) presents the determined regression equation of the electric power input depending on the pyrolyzer's achieved temperature.

(18) $$E=0.21+0.0083x+4.5\cdot 10^{-6}x^{2}$$

where: E − Value of electricity input, kWh.

The coefficient of determination R^2^ is equal to 0.9941. When the current energy E was converted to MJ, equation (19) assumed the value:

(19) $$\text{Ee}=0.756+0.02988x+1.62\cdot 10-5x^{2}$$

where: Ee - the value of the input of electricity energy, MJ.

#### 3.2.2 Energy relations

An energy assessment of the pyrolysis process was performed by comparing energy inputs and outputs relative to the input feedstock. It was considered that the obtained results are related, among other things, to the design and operating parameters of the reactor used in the study and the amount of raw material gasified.

The total energy contained in the gas obtained from the pyrolysis of 6.0 kg of wheat straw pellets in the reactor was determined from the following equations:

- (4) describing the effect of the process temperature on the mass contribution of the synthesis gas to the total mass of products obtained from pyrolysis, %_mass_,
- (10) describing the effect of the process temperature on the higher heating value of the gas produced in the process, MJ·kg^−1^. After converting the individual values from the equations adopted for the analysis and taking into account the batch mass of the pellet, an equation (20) was obtained, which describes the effect of process temperature on changes in the energy contained in the gas produced from the wheat straw load entering the reactor:

(20) $$\text{Eg}=-\;0.5675+6.086\cdot 10^{-3}x+6.14314\cdot 10^{-5}x^{2}+7.61\cdot 10^{-6}x^{3},\text{MJ}$$

where: Eg - energy contained in pyrolysis gas, MJ.

The changes in the energy content of biochar were determined from equation (16) when multiplied by the value of the weight of the loaded raw material the mathematical formula (21) describing the effect of reactor temperature on the energy contained in the pyrolytic liquid was kept.

(21) $$\text{Eb}=109.932\;-\;0.2376x+3.03\cdot 10^{-4}x^{2}\;-\;1.448\cdot 10^{-7}x^{3}\text{,}$$

where: Eb is the energy contained in the biochar, MJ.

**Fig. 11:**
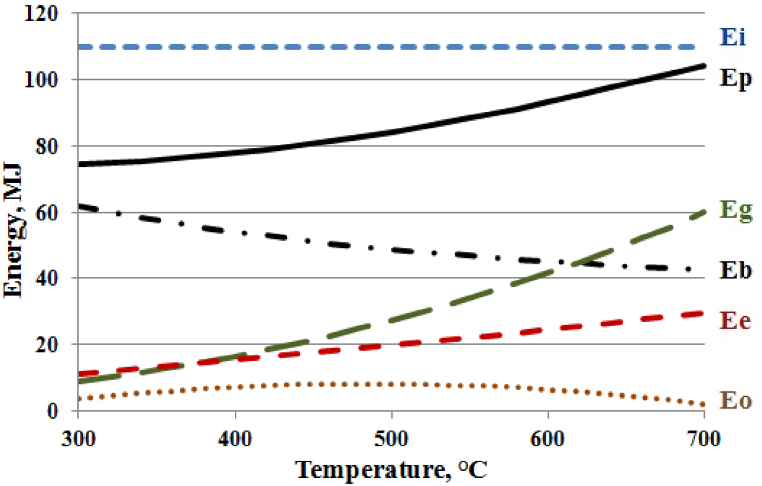
Effect of reactor temperature on electricity input and energy of pyrolysis products: Ee - electricity input, Eo - energy contained in the pyrolysis liquid, Eg - energy contained in the gas, Eb - energy contained in the biochar, Ei - energy contained in the raw material, Ep - total energy of the pyrolysis products, MJ.

Using mathematical (17), (19), (20), (21), which describe: Eo - the energy contained in the pyrolysis liquid, Ee - the value of electricity input, Eg - the energy contained in the pyrolysis gas, and Eb - the energy contained in the biochar, lines were drawn in the graph to describe the effect of reactor temperature on changes in the energy contained in the pyrolysis products in relation to the total mass of the raw material consumed. The graph is shown in Fig. 11.

There is also presented line Ep in Fig. 11, named - the total energy of pyrolysis products. This line was detrmined using equation (22), which was calculated by adding together formulas describing the energies contained in the pyrolysis products: pyrolysis liquid (17), synthesis gas (20), and biochar (21).

(22) $$\text{Eb}=85.227\;-\;0.131634x+2.1483\cdot 10^{-4}x^{2}\;-\;6.87\cdot 10^{-8}x^{3}$$

### 3.3 Discussion

#### 3.3.1 Pyrolytic gas

During straw pyrolysis, the proportion of gas produced relative to the mass of the feedstock at a reactor temperature of 700 °C was significant, at 52.7 % (Fig. 4). Since the energy potential of the pyrolysis gas obtained from the wheat straw at this reactor temperature was found to be twice the amount of electricity used to produce this gas, there is the possibility of recovering at least a significant proportion of the electricity used. It is also possible to obtain additional thermal energy through cogeneration with electricity production or to obtain thermal energy alone, with a much higher value than the consumed electricity.

If classic Power-to-Gas technology via water electrolysis were used instead of straw pyrolysis, the energy potential of the resulting gas - hydrogen - would be less than the electricity consumed in water electrolysis. Alkaline Electrolyzer efficiency ranges from 47 to 82, and when using Polymeric membrane (PEM) fuel cells for this purpose, the energy efficiency is 62–90 % [41]. A prerequisite for using the obtained gas as an energy-accumulating material is that it can be stored in a tank. This applies to the hydrogen and oxygen obtained from electrolysis and the gas produced during pyrolysis. However, the gas obtained in the wheat straw pyrolysis experiments may prove difficult to store [42] due to the composition of the cooled gas, which contains unnecessary carbon dioxide, low-energy carbon monoxide, methane, and hydrogen. These components are challenging to store in tanks. Although the changes in the gas composition and the increase in its energy properties were positively influenced by the rising reactor temperature (Fig. 5), the carbon dioxide content decreased only from 59.2 to 19.1 % vol. The carbon monoxide content remained at the same level between the initial and final temperatures of the experiments (33.9 %–32.4 % vol) but decreased between 400 and 600 °C. The most significant increases in synthesis gas components were observed for methane and hydrogen. Methane content increased from 6.8 % vol (300 °C) to 26.8 % vol (700 °C), and hydrogen, which appeared in the gas only at 400 °C with a content of 3.6 % vol, reached its highest content of 21.7 % vol at 700 °C. These changes in the proportions of individual gases in the pyrolysis gas volume, resulting from raising the reactor temperature, led to a decrease in its density from 1.521 to 0.921 kg m_N_^−3^ (Fig. 6a) and more than a fourfold increase in its heat of combustion, from 4.47 to 17.94 MJ kg^−1^ (Fig. 6b).

The generated pyrolytic gas could possess a higher energy value and occupy less space if it underwent purification and refinement. Carbon dioxide, having no intrinsic energy value, becomes unnecessary ballast in the pyrolytic gas due to its significant weight and volume. As a result, it should be entirely removed from the gas. One effective method for CO2 removal from gases involves membrane technologies [43]. Additionally, ongoing research is exploring novel approaches to capture and separate carbon dioxide from pyrolytic gas, utilizing waste heat [44]. Furthermore, the CO_2_ content can be reduced during the pyrolysis process by employing catalysts like CaO and Ni–CaO [45,46].

Energy storage technologies in the form of gas (Power-to-Gas) are prompting researchers to look for other ways to refine energy gases, for example, by converting them to one type of gas. In the case of pyrolytic gas, this could be its methanation [47]. The hydrogen in pyrolytic gas can pose a problem when stored in a tank and flow uncontrollably through tank walls due to its particle size. Ongoing research on the methanation of hydrogen-containing gas using carbon dioxide, also contained in pyrolysis gas, indicates the possibility of joint conversion of these gases to methane [[48], [49], [50], [51]].

During the pyrolysis, the temperature of the gas leaving the reactor was more than twice as low as the reactor temperature (Table 3). Its value was not only the result of the heat imparted to the reactor but also the effect of the thermo-chemical transformations and the limitations associated with the heat and mass exchange processes occurring inside the reactor [34,35]. The temperature inside the reactor and the pyrolysis process may have been influenced by the size of the particles and the dose of heat the biomass takes on inside the reactor. The gas leaving the reactor only at its temperature of 700 °C had a temperature sufficient for CO_2_ methanation, especially in the presence of selective catalysts [51]. Analysis of the results from the performed experiments also indicated that a way to reduce the CO_2_ content of the pyrolysis gas, even without using catalysts, could be to raise the temperature in the reactor above 700 °C (Fig. 5).

The generation of electricity from pyrolysis gas would be carried out by a generator driven by an internal combustion engine. The transformation efficiency of the energy contained in the gas depends on the current load of the generator and reaches up to 28 % [52]. The energy efficiency of using a hydrogen fuel cell for this purpose is between 30 and 60 % [41].

#### 3.3.2 Biochar

The mass of the produced biochar decreased with increasing temperature during pyrolysis, reaching approximately 27 % relative to the raw material used (Fig. 4). It was similar to the results from previous studies [18,19]. As the process temperature rose, the carbon content in the biochar increased, going from 65.7 % by mass at 300 °C to 73.1 % by mass at 600 °C (Table 5). Similar results regarding changes in carbon content in biochar derived from wheat straw were observed in earlier studies [18]. TGA analysis of the biochar indicated that process temperature had an effect on the increase in ash content in the remaining biochar, rising from 16.6 % by mass to 22.5 % by mass (Table 5). However, this increase in ash content is primarily a result of reduced biochar yield as the process temperature increased, along with the loss of organic matter. The rise in ash content due to the shrinkage of solid residue under higher process temperatures may explain the observed increase in minerals in the biochar [15,18,19]. Due to the higher process temperatures, this loss of organic matter reduced the overall fuel content in biochar, from 83.4 % by mass at 300 °C to 77.5 % by mass at 700 °C (Table 5). This is primarily due to a decrease in volatile fuel content, dropping from 26.8 % by mass at 300 °C to 7.1 % by mass at 700 °C. Proximate analysis indicates that this is also linked to the reduction of oxygen and hydrogen content in the solid residues (Fig. 7). The higher pyrolysis temperature increased gas production and decreased biochar yield and carbon content within biochar (Fig. 4, Fig. 7). The carbon content in the used straw was 47.40 %, next decreased to 20.23 % in relation to the used feedstock in the biochar obtained at 700 °C. When considering the yield of biochar from pyrolysis of waste biomass for agricultural purposes, it's essential to account for the carbon sequestered in the soil through the introduction of biochar. This quantity should not fall below the carbon footprint resulting from the combined energy inputs allocated to fertilizer and machinery production and plant cultivation, especially the straw used in the pyrolysis process. Adhering to this approach will ensure greenhouse gas-neutral agricultural production and the efficient use of agricultural waste biomass for energy purposes.

#### 3.3.3 Liquid

The pyrolysis liquid contains a significant amount of water. Purification of the gas substance before cooling could effectively separate the tar-oil fraction from the water, thus purifying it. Alternatively, the pyrolytic liquid can undergo an extraction process to isolate its individual components [29]. The separated tar-oil fractions can serve as a liquid energy source in a boiler or can be further processed into other fuels like ethanol and diesel, as well as chemicals, through hydrodeoxygenation [53]. Consideration can also be given to improving liquid quality through pyrolysis with different feedstocks [54].

Table 6 and Fig. 9 illustrate that a reactor temperature of 500 °C marked a critical point for both the capacity of the pyrolytic liquid and the amount of oil-tar fraction and energy it contained. Beyond this temperature, there was a noticeable decrease in liquid volume and the content of the oil-tar fraction. Although the liquid retained a tar-oil fraction with significant calorific value, its high water content may render direct use as a fuel impractical or uneconomical. The percentage of water content in the pyrolytic liquid ranged from 78.3 % to 95.2 %, with the lowest value was achieved at 500 °C.

Based on the product of the volume of the pyrolytic liquid as a proportion of the feedstock charge (see Fig. 4), the content of the tar-oil fraction within the pyrolytic liquid, and the Higher Heating Value (HHV) of this fraction, the total internal energy contained in the liquid volume was calculated. The results of this calculation are presented in Fig. 9.

#### 3.3.4 Energy

Fig. 11 illustrates the most significant decrease in the energy content of the pyrolysis products occurring at a reactor temperature of 300 °C. This situation can be explained by referring to Fig. 7, where a notable decrease in oxygen, carbon, and hydrogen content in the biochar at this temperature is evident. Consequently, a substantial amount of water (as shown in Fig. 5 and Table 6) and significant quantities of carbon dioxide (as seen in Fig. 6) are formed. Neither of these products carries useful energy. With further temperature increase, driven by higher electricity consumption, the energy stored in the products, particularly in the pyrolysis gas, becomes more pronounced. At approximately 374 °C, the energy accumulated in the resulting gas matches the electricity required to produce it. By 700 °C, the energy in the gas surpasses twice the value of the electrical energy input into the reactor.

Fig. 11 also demonstrates that elevating the reactor temperature could potentially diminish the energy properties of the pyrolysis liquid and might even lead to the complete absence of the oil fraction in it (as indicated in Fig. 4 and Table 6).

## 4 Summary

The increase of temperature in screw pyrolyzer increased electricity consumption, but the amount of gas produced and its energy value increased much faster, even twice the amount of energy consumed. Therefore, it can be concluded that the installation that uses electricity and a screw reactor to pyrolyze straw for electricity storage is an innovative approach to power-to-gas technology. The amount of gas obtained in the planned installation and its energy value can help reproduce the electricity consumed in its production and obtain additional heat energy.

Studies have shown that the plant containing an electrically powered reactor for straw pyrolysis is preferable for farms because, in addition to gas production, it produces a significant amount of biocarbon for soil enrichment and sequestration of atmospheric carbon.

A comparison between the energy efficiency of storing electricity using an electrolyser and a fuel cell and water as a feedstock and the energy efficiency of storing electricity using straw pyrolysis and an internal combustion engine-driven generator will be possible once experiments have been conducted. The balance sheet should include all energy inputs for raw material preparation, purification, and upgrading pyrolysis gas and gases from electrolyse.

The pyrolytic liquid produced by the pyrolysis of wheat straw contains a massive amount of water. Although it also contains tar oil as a component with a significant calorific value, using it as fuel may not be economical.

## Funding and acknowledgments

This work was supported by the REFRESH - Research Excellence For REgion Sustainability and High-tech Industries, Registration Number - CZ. 10.03.01/00/22_003/0000048.

## Data availability

Data will be made available on request.

## CRediT authorship contribution statement

**Jerzy Chojnacki:** Writing – original draft, Validation, Methodology, Formal analysis, Data curation, Conceptualization. **Jan Kielar:** Writing – review & editing, Validation, Resources, Methodology, Data curation, Conceptualization. **Jan Najser:** Writing – review & editing, Validation, Resources, Methodology, Conceptualization. **Jaroslav Frantík:** Validation, Methodology. **Tomáš Najser:** Validation, Supervision, Methodology, Data curation. **Marcel Mikeska:** Methodology, Data curation. **Błażej Gaze:** Validation, Conceptualization. **Bernard Knutel:** Validation, Conceptualization.

## Declaration of competing interest

The authors declare that they have no known competing financial interests or personal relationships that could have appeared to influence the work reported in this paper.
